# A Novel ALAS2 Missense Mutation in Two Brothers With Iron Overload and Associated Alterations in Serum Hepcidin/Erythroferrone Levels

**DOI:** 10.3389/fphys.2020.581386

**Published:** 2020-11-12

**Authors:** Acaynne Lira Zidanes, Giacomo Marchi, Fabiana Busti, Alessandro Marchetto, Elisa Fermo, Alejandro Giorgetti, Alice Vianello, Annalisa Castagna, Oliviero Olivieri, Paola Bianchi, Domenico Girelli

**Affiliations:** ^1^Section of Internal Medicine, Department of Medicine, University of Verona, Verona, Italy; ^2^EuroBloodNet Referral Center for Rare Disorders of Iron Metabolism, University Hospital of Verona, Verona, Italy; ^3^Department of Biotechnology, University of Verona, Verona, Italy; ^4^Hematology and Pathophysiology of Anemias Unit, Istituto di Ricovero e Cura a Carattere Scientifico (IRCSS) Ca’ Granda Foundation, Policlinico Milano, Milan, Italy

**Keywords:** XLSA, ERFE, hepcidin, *ALAS2* gene, next-generation sequencing, *in silico* modeling, iron-loading anemias

## Abstract

Iron loading anemias are characterized by ineffective erythropoiesis and iron overload. The prototype is non-transfusion dependent ß-thalassemia (NTDT), with other entities including congenital sideroblastic anemias, congenital dyserythropoietic anemias, some hemolytic anemias, and myelodysplastic syndromes. Differential diagnosis of iron loading anemias may be challenging due to heterogeneous genotype and phenotype. Notwithstanding the recent advances in linking ineffective erythropoiesis to iron overload, many pathophysiologic aspects are still unclear. Moreover, measurement of hepcidin and erythroferrone (ERFE), two key molecules in iron homeostasis and erythropoiesis, is scarcely used in clinical practice and of uncertain utility. Here, we describe a comprehensive diagnostic approach, including next-generation sequencing (NGS), *in silico* modeling, and measurement of hepcidin and erythroferrone (ERFE), in two brothers eventually diagnosed as X-linked sideroblastic anemia (XLSA). A novel pathogenic *ALAS2* missense mutation (c.1382T>A, p.Leu461His) is described. Hyperferritinemia with high hepcidin-25 levels (but decreased hepcidin:ferritin ratio) and mild-to-moderate iron overload were detected in both patients. ERFE levels were markedly elevated in both patients, especially in the proband, who had a more expressed phenotype. Our study illustrates how new technologies, such as NGS, *in silico* modeling, and measurement of serum hepcidin-25 and ERFE, may help in diagnosing and studying iron loading anemias. Further studies on the hepcidin-25/ERFE axis in additional patients with XLSA and other iron loading anemias may help in establishing its usefulness in differential diagnosis, and it may also aid our understanding of the pathophysiology of these genetically and phenotypically heterogeneous entities.

## Introduction

Iron loading anemias are anemias characterized by ineffective erythropoiesis and iron overload (Camaschella and Nai, 2016). They include non-transfusion dependent ß-thalassemia (NTDT) (Musallam et al., 2012), congenital sideroblastic anemias (Fujiwara and Harigae, 2019), congenital dyserythropoietic anemias (Iolascon et al., 2013), some hemolytic anemias, and myelodysplastic syndromes (Tanno and Miller, 2010; Camaschella and Nai, 2016; Brissot et al., 2018). X-linked sideroblastic anemias (XLSA), which can be referred also to the group of atypical microcytic anemias (Donker et al., 2014), can be suspected starting from simple blood exams showing microcytic anemia with paradoxically high ferritin after easily discarding more frequent conditions such as thalassemia and anemia of inflammation (Camaschella, 2013; Donker et al., 2014). Regarding the pathogenesis of iron overload in iron loading anemias, the hepcidin/erythroferrone (ERFE) axis seems to play a crucial role, also representing a promising new therapeutic target (Arezes et al., 2020). Hepcidin is the master regulator of systemic iron homeostasis, which acts by controlling intestinal iron absorption and macrophage iron recycling through the inhibition of the iron exporter ferroportin (Ganz, 2011; Girelli et al., 2016). The recently described hormone ERFE is produced by erythroblasts in response to erythropoietin (EPO) and acts by suppressing hepcidin, thereby increasing iron absorption and mobilization for erythropoiesis demand (Kautz et al., 2014; Coffey and Ganz, 2018). ERFE, likely in addition to other mediators, is thus thought to contribute to secondary iron overload in iron loading anemias. With the advent of next generation sequencing (NGS) techniques, genes responsible for sideroblastic anemias are often included in panels designed for diagnosing hereditary anemias, allowing for detection of an increasing number of cases, reducing misdiagnosis, and highlighting the phenotypic variability of this group of disorders.

X-linked sideroblastic anemia (XLSA; OMIM 301300) is caused by loss-of-function mutations in the erythroid-specific 5-aminolevulinate synthase gene (*ALAS2*) (Cotter et al., 1994). *ALAS2* gene encodes for mitochondrial 5-aminolevulinate synthase (ALAS2), the first enzyme in heme biosynthetic pathway in erythroid cells (Bishop et al., 1990; Cox et al., 1990). ALAS2 catalyzes the condensation of glycine and succinyl-CoA into 5-aminolevulinic acid (ALA), using pyridoxal 5′-phosphate (PLP) as a cofactor (Ducamp et al., 2011). To date, more than 80 different mutations in *ALAS2* gene have been reported in patients with XLSA (Ducamp and Fleming, 2019) (Human Genome Mutation database^1^). Most of these are missense mutations located within a conserved region (encoded by exons 5–11), leading to a reduced ALAS2 activity and/or stability (Ducamp and Fleming, 2019). Mutations in the *ALAS2* regulatory region, such as the promoter and intron 1, have also been reported, resulting in decreased *ALAS2* expression (Bekri et al., 2003; Campagna et al., 2014).

XLSA is the most common subtype of Congenital Sideroblastic Anemia (CSA) and typically affects hemizygous males, who often show a mild to moderate anemia since childhood with complications related to iron overload in adulthood. The anemia is hypochromic and microcytic in males, with a mean corpuscular volume (MCV) between 60 and 70 fL and accompanying laboratory signs of iron overload, i.e., high ferritin and transferrin saturation (Bergmann et al., 2010), but almost always normocytic or macrocytic in females. However, severity varies widely depending on the effect of the mutation in ALAS2 protein and additional factors. The phenotypic expression of XLSA is variable between families and also within relatives of a given affected family (Cazzola and Malcovati, 2015; Brissot et al., 2018). Although patients with XLSA are predominantly males, because of hemizygosity of the X-linked defect, many cases of female patients with the heterozygous *ALAS2* mutation have also been reported (Fujiwara and Harigae, 2019), and this is usually due to an age-related skewing of X chromosome inactivation. Additional genetic or somatic mutations and environmental factors may contribute to phenotypic variability (Donker et al., 2014). For example, co-inheritance of HFE mutations may worsen the degree of iron overload in hemizygous males (Cotter et al., 1999). XLSA treatment is focused on two aspects: anemia and iron overload. Most patients are not transfusion-dependent; however, they may develop a transfusion need with increasing age. Anemia and ineffective erythropoiesis often benefit from pyridoxine treatment, although pyridoxine-responsiveness is lower in the case of iron overload (Cotter et al., 1999). Low-regimen phlebotomies (e.g., 200–250 mL every 2 weeks) or iron chelating agents are used in the case of iron overload (Cazzola and Malcovati, 2015).

This report illustrates how new technologies, such as NGS and measurement of serum hepcidin-25 and ERFE, may help in diagnosing and studying iron loading anemias. We describe the paradigmatic case of a male proband diagnosed with XLSA through NGS, who had a novel *ALAS2* missense mutation. His brother also carried the same mutation; however, his phenotypic expression was slightly different. We also provide the *in silico* modeling of the novel mutation and measurements of serum hepcidin-25 and serum ERFE as possible tools for better understanding the pathophysiology of iron overload in XLSA.

## Methods

### Patients

Informed consent was obtained before conducting the experimental analysis. All the procedures performed in this study were in accordance with the ethical standards of our Ethical Committee and with the 1964 Helsinki declaration and its later amendments.

DNA samples were collected from both patients, who gave written informed consent to DNA analysis, according to study protocols approved by the local Ethical Committee.

### *ALAS2* Gene Analysis

Genomic DNA was extracted from peripheral blood leukocytes through salting out method (Miller et al., 1988) using the Wizard Genomic DNA purification kit (Promega). The DNA extraction was performed according to the manufacturer’s instructions.

The DNA sample of the proband was analyzed on an NGS-targeted panel SureDesign software (Agilent Technologies, Santa Clara, United States) containing 40 genes associated with congenital hemolytic anemia and modifier genes (Rotordam et al., 2019). Libraries were obtained by HaloPlexHS Target Enrichment System Kit and sequenced on a MiSeq platform (Illumina, San Diego, United States). Targeted filtering and annotation of protein-changing variants were performed using the wANNOVAR web tool^2^.

The mutation identified was confirmed by Sanger method (ABI PRISM 310 Genetic Analyzer, Applied Biosystems, Warrington, United Kingdom) using the Big Dye Terminator Cycle Sequencing Kit (Applied Biosystems, Warrington, United Kingdom).

### Sequence Analysis and *in silico* Modeling

*In silico* predictions of missense variants’ pathogenicity was performed using SIFT (Kumar et al., 2009) and Polyphen-2 (Adzhubei et al., 2010) bioinformatics tools. Reviewed ALAS2 sequences from different species were retrieved from the UniProtKB/Swiss-Prot database and aligned using the MUSCLE (Edgar, 2004) program for multiple sequence alignments. Conservation analysis and alignment visualization were performed by Jalview software (version 2)^3^ (Waterhouse et al., 2009) and they are available from IronGenes website^4^.

The structural analysis of the missense variants was made based on the available human ALAS2 crystallographic structure (Bailey et al., 2020) (PDB accession code: 5QQQ, crystallographic resolution: 1.93 Å).

The Consurf server (Ashkenazy et al., 2016) was used to map conservation features on the structure. The prediction of the putative effects of the variants in the structure/function of the protein was performed also by visual inspection using the Chimera program. The wild-type residues and the modeled mutant were included in the publicly accessible IronGenes database^5^.

### Hepcidin and Erythroferrone Measurement

Hepcidin measurement was performed using an updated and validated Mass-Spectrometry (MS)-based assay (Castagna et al., 2009). This analysis allowed the quantification of the mature bioactive circulating isoform (hepcidin-25) and two smaller isoforms (hepcidin-24 and hepcidin-20), using a chromatography-tandem mass spectrometry (LC-MS/MS) approach (van der Vorm et al., 2016). Hepcidin-25 synthetic standards (the native and the isotopic labeled internal standard), and standards for hepcidin-24 and hepcidin-20 isoforms, were purchased from Peptide International (Lousiville, United States). Briefly, an internal standard was added in all samples, and the calibration curve was created. Blank serum, deprived of hepcidin, was prepared using charcoal treatment. The calibration curve was prepared with the blank serum and a known concentration of standards of each hepcidin isoform. Samples were treated by solid-phase extraction using Oasis hydrophilic-lipophilic balanced reversed-phase (HLB) cartridges (Waters, Italia). High-performance LC was performed using an X-Terra MS C18 2.5 mm column (Waters, Italia), and detection was obtained using a Triple Quad LC-MS/MS (Agilent Technologies). The results were evaluated according to previously obtained reference ranges for males and females at different ages (Traglia et al., 2011).

Erythroferrone analysis was performed using the Erythroferrone IE^TM^ ELISA kit (Intrinsic Lifesciences-The BioIron Company^TM^), a double monoclonal antibody sandwich ELISA method, according to manufacturer instructions. The concentration of human ERFE was obtained from the mean absorbance of the standard curve. The reference range (0.32–1.80 ng/mL) was obtained from a recent publication that evaluated ERFE levels using the same ELISA kit in 78 males with median age 47 years (Appleby et al., 2020).

### Magnetic Resonance Imaging

Organ iron distribution was not-invasively studied with Magnetic Resonance Imaging (MRI), according to Gandon’s protocol (Gandon et al., 2004) to define liver iron content (LIC) and spleen iron content (*SIC*) and with T2/T2^∗^ sequences (Garbowski et al., 2014). Spleen volume was estimated based on a three-axis approach (Prassopoulos et al., 1997).

## Results

The proband was a 56 years-old male, referred to our Center because of a microcytic anemia known since childhood and hyperferritinemia. Personal history and physical examination revealed allergic asthma treated with inhalers, obesity (BMI 33 Kg/m^2^), hypertension, and splenomegaly. He only received five units of packed red blood cells in his life during a hospitalization for a transient severe drop of Hb levels. First-level laboratory analysis showed Hb 103 g/L, MCV 73.3 fL, ferritin 1,493 ng/mL, transferrin saturation 63%; no signs of hemolysis, chronic hepatitis, or inflammation were detected. The bone marrow smear showed erythroid hyperplasia with dyserythropoiesis and 2–3% of blasts, ringed sideroblasts were 8–10%. A review of historical complete blood counts (CBCs) in the proband showed Hb values around 110–120 g/L. He had a younger brother who also had microcytosis and low to normal Hb levels (around 130 g/L in historical CBCs series).

Patients’ characteristics are reported in Table 1, including laboratory and instrumental data at the time of diagnosis.

**TABLE 1:** Clinical characteristics of the two brothers.

Laboratory data	Proband	Younger brother	(Reference range)
	(M, 56 years old)	(M, 53 years old)	
Hb (g/L)	103	134	(130–170)
RBCs (×10^12^)	5.2	6.14	*(4.50–5.80)*
MCV (fL)	73.3	69.2	(79–96)
MCH (pg)	19.8	21.8	(27–33)
Reticulocytes (×10^9^)	54	/	(27–99)
WBCs (×10^9^)	5.9	8.3	(4–10)
PLTs (×10^9^)	286	305	(150–400)
Ferritin (μg/L)	1,493	890	(30–300)
Transferrin saturation (%)	63	28.6	(20–50)
GOT (U/L)	38	24	(5–40)
GPT (U/L)	58	40	(10–65)
Bilirubin (mg/dL)	0.8	0.3	*(0.0–1.2)*
Creatinine (mg/dL)	0.7	0.9	*(0.6–1.4)*
Folate (ng/mL)	> 20	2.7	*(3.8–20)*
Vitamin B12 (pg/mL)	499	483	(197–866)
Ringed sideroblasts in bone marrow	8–10%	n.a.	(Absence)
Erythroferrone (ng/mL)	75.51	14.47	*(0.32–1.80)*
Hepcidin-25 (nM/L)	27.65	10.34	*(1.8–9.2)*
Hepcidin:Ferritin ratio (pM/μg)	18.5	11.6	*(20.9–25.3)*
MRI-LIC (liver iron content) (μM/g)	295	96	*(*<*36)*
MRI-SIC (spleen iron content) (μM/g)	134	127	(Unvalidated)
MRI-Pancreas T2/T2* (ms)	24.6	n.a.	*(*>*26)*
MRI-Heart T2/T2* (ms)	43	n.a.	*(*>*20)*
Spleen volume estimated on MRI (mL)	562	265	(110–340)
*HFE* sequencing	Negative for C282Y and H63D mutations	Negative for C282Y and H63D mutations	(No mutations)
*ALAS2* sequencing	Novel mutation (c.1382T>A p.Leu461His)	Novel mutation (c.1382T>A p.Leu461His)	(No mutations)
Transfusion-dependency	No	No	
Number of packed red blood cells transfused in life	5	0	
Comorbidities	Allergic asthma, obesity, hypertension	None	
Subsequent treatment	Pyridoxine, folate, deferasirox	Pyridoxine, folate, low regimen phlebotomies	
Outcome	Iron-depletion; Hb 103 → 114 g/L, MCV 73 → 75 fL	Iron-depletion; Hb 134 → 137 g/L, MCV 69 → 75 fL	

### *ALAS2* Mutation and *in silico* Modeling

**FIGURE 1:**
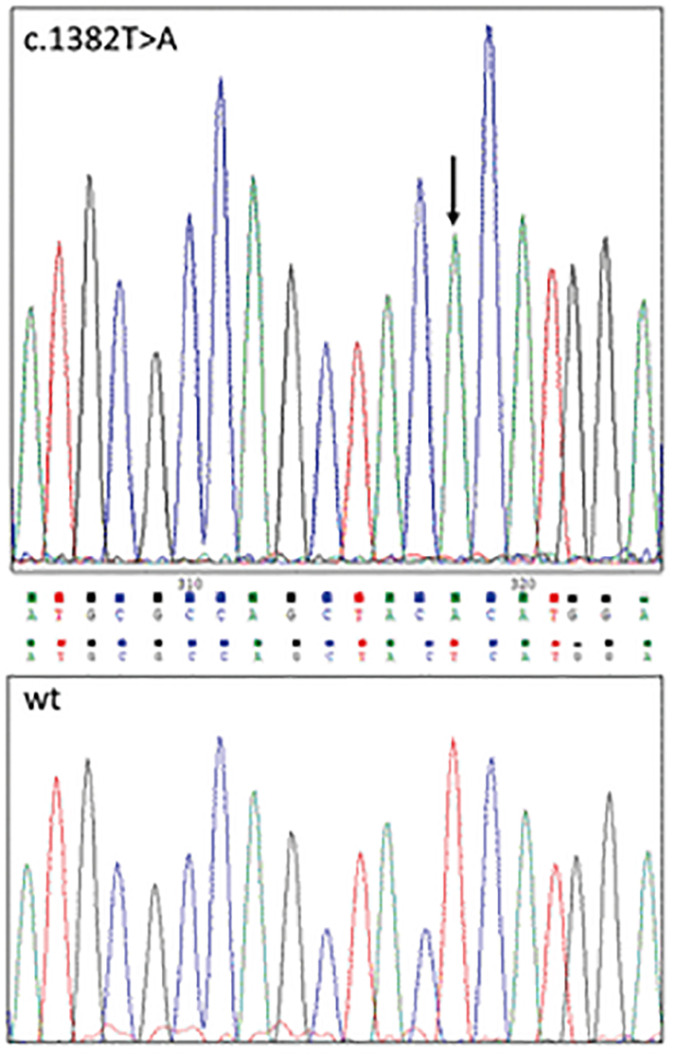
The ALAS2 c.1382T>A mutation confirmed by Sanger sequencing.

A novel missense mutation in *ALAS*2 gene (c.1382T>A, p.Leu461His, NM_000032.5), located in exon 9 was identified in the proband by targeted NGS, and confirmed by Sanger sequencing in both the proband and the brother (Figure 1). No other pathogenic variants associated with congenital anemias were detected.

The new mutation was not been previously reported in XLSA patients and was predicted to be probably damaging using five predictive tools: Mutation Taster^6^, Polyphen-2^7^, SIFT^8^, MutPred^9^, and SNPs&GO^10^. Splice site prediction tools showed no evidence of slicing site abnormalities (NetGene2^11^; NNSplice^12^; MutPred Splice^13^).

The variant was neither found in ExAC nor gnomAD and classified as likely pathogenic according to ACMG Standards and Guidelines (Richards et al., 2015).

Targeted NGS analysis also excluded concomitant presence of mutations in HFE gene associated with hemochromatosis.

**FIGURE 2:**
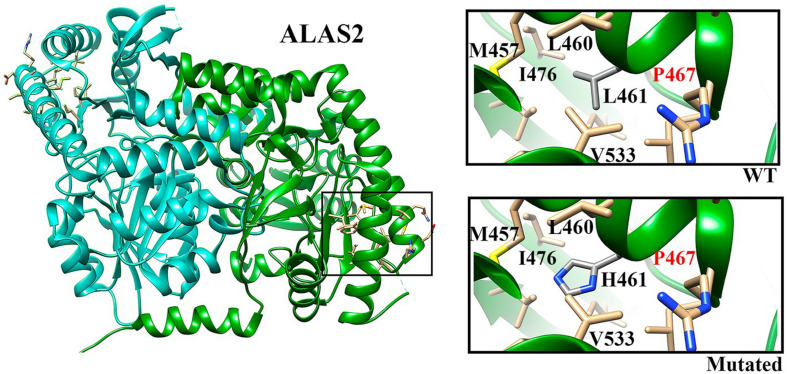
ALAS2 structure. Chains A and B are indicated in green and cyan, respectively. The selected region (square) indicates the localization of L461 residue. In the insights we show the WT residue (upper) and the mutated residue (lower) with the closest neighbors.

In position 461, the leucine residue appears well conserved (58.1%) in our multiple sequence alignment (see footnote 4). Moreover, the presence of a hydrophobic residue (Leu, Ala, and Val) at that position is ensured for more than 90% of the sequences, indicating the need of a hydrophobic residue able to stabilize that protein region locally. Indeed, our *in silico* analysis of the amino acids around the mutated residue points in this direction. Figure 2 shows that Leu461 is surrounded by a bunch of hydrophobic residues, i.e., L460, M457, V533, and I476 among others, and that its mutation into a histidine residue may hamper the formation of this hydrophobic network (Figure 2) (see footnote 5). Indeed, the I476 residue has been shown to reduce the enzymatic activity when mutated into Asn, likely by altering the local folding of the mutant enzyme (Cotter et al., 1992). Similarly to the I476N mutation (rs137852299), the L461H mutation introduces a polar residue in a hydrophobic environment. We therefore cannot exclude a similar effect on the local folding of the enzyme.

### Serum Hepcidin and Erythroferrone

High hepcidin-25 levels were found in both patients, especially in the proband (27.65 vs. 10.34 nM/L, normal range 1.8–9.2 nM/L). Ferritin levels were increased in both patients and higher in the proband (1,493 vs. 890 μg/L, normal range 30–300 μg/L). The hepcidin:ferritin ratio was decreased in both patients (18.5 and 11.6, respectively, normal range 20.9–25.3). ERFE levels in the proband, who had the more expressed phenotype, were markedly higher than reference range (75.51 ng/mL, reference range 0.32–1.80 ng/mL) and about five times higher than those of his brother (14.47 ng/mL) (Table 1).

### Magnetic Resonance Imaging

**FIGURE 3:**
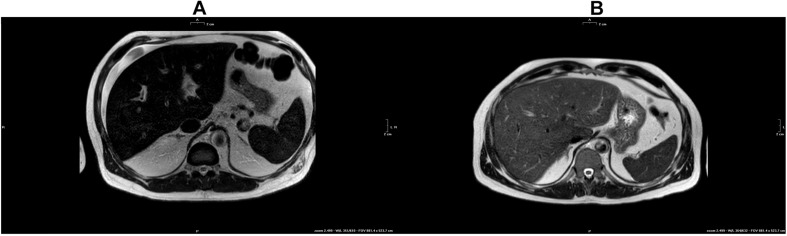
**(A)** T2-w MRI of the proband. **(B)** T2-w of the younger brother.

In the proband, Magnetic Resonance Imaging (MRI) demonstrated a significant iron accumulation in liver (LIC 295 μM/g), and a mild accumulation in spleen (*SIC* 134 μM/g) and pancreas (T2/T2^∗^ 24.6 ms) (Figure 3A and Table 1), whereas no accumulation was detected in heart (T2/T2^∗^ 43 ms). The younger brother had a mild accumulation in liver (LIC 96 μM/g) and spleen (*SIC* 127 μM/g) (Figure 3B and Table 1). Only the proband had splenomegaly (562 vs. 265 mL).

## Discussion and Conclusion

Our targeted NGS panel analysis revealed a novel *ALAS2* missense mutation c.1382T>A (p.Leu461His) in exon 9 in the two brothers. Although Sanger sequencing is usually the first choice in patients presenting with classical features of X-linked sideroblastic anemia due to low costs, we choose performing a NGS panel analysis due to our experience on a not negligible prevalence of digenic inheritance in iron overload disorders (Badar et al., 2016), as well as because of the slightly different phenotype in the two brothers. Leu461 is located in an α-helix, which in turn is located in the central catalytic domain, the most evolutionary conserved domain of ALAS2. Indeed, our sequence alignment analysis revealed that Leu461 (or a hydrophobic residue) is highly conserved across different species. Previously described pathogenic mutations were found in the same highly conserved domain of the protein (Cotter et al., 1992). Indeed, our *in silico* modeling showed that the mutant residue (His) is bigger than the wild type residue (Leu), and with very different physicochemical properties. The inclusion of a polar/charged amino acid in a highly hydrophobic environment (Figure 2) (see footnote 5) could, with high probability, cause alterations in the local folding of the protein (alpha-helix structure) by disrupting the local hydrophobic core network of interactions.

Iron homeostasis in the two affected brothers was studied, linking biochemical parameters, serum hepcidin-25 and ERFE, MRI organ iron distribution, and clinical characteristics. Both patients had a mild-to-moderate iron overload, with some differences. The proband had a more expressed phenotype with lower Hb and higher ferritin, TSAT, LIC and spleen volume. His ERFE and hepcidin levels were higher compared to reference range and to the younger brother. However, when hepcidin was studied in relation to ferritin levels, the hepcidin:ferritin ratio was decreased in both patients, indicating that hepcidin levels were not as high as they would be expected for the ferritin levels. Indeed, since hepcidin is physiologically regulated by body iron stores, the usefulness of hepcidin:ferritin ratio is to assess whether or not hepcidin production is appropriate for the degree of iron overload. Surprisingly, the younger brother, who had the milder phenotype and milder iron overload, had the lower hepcidin:ferritin ratio. According to current hypothesis, in iron loading anemias, erythroid signals override signals from the replete stores, causing and perpetuating iron overload, with ERFE being the major candidate erythroid regulator of hepcidin production (Kautz and Nemeth, 2014; Camaschella and Nai, 2016). In humans it has been showed that blood loss or EPO administration increase serum ERFE concentrations, and that patients with both NTDT and transfusion-dependent β-thalassemia have very high serum ERFE levels, which decrease after blood transfusion (Ganz et al., 2017). ERFE levels in our two patients with XLSA were quite higher than normal, resembling levels found in NTDT patients (Ganz et al., 2017). This suggests the presence of a significant erythroid stimulus affecting iron metabolism notwithstanding a relatively mild XLSA phenotype. Nonetheless, further studies are needed in additional patients with XLSA, other sideroblastic anemias, and other iron loading anemias.

Our study has the obvious limitation that, given the rarity and the molecular heterogeneity of mutations in *ALAS2* gene, no other cases carrying this mutation have been described so far. The different clinical severity observed in the two brothers raised the possibility of concomitant causes of anemia or iron overload, here excluded by targeted-NGS panel analysis. Furthermore, it must be taken into account that environmental factors, like obesity in the proband, may have influenced the phenotype.

In conclusion, our report illustrates how new methods, like NGS panels, hepcidin-25 and ERFE measurement, may help in differential diagnosis of iron loading anemias. Further studies on the hepcidin-25/ERFE axis in additional patients with XLSA and other iron loading anemias may help in establishing its usefulness in the differential diagnosis as well as to better understand pathophysiology of these genetically and phenotypically heterogeneous entities.

## Data Availability Statement

The sequencing data has been deposited into the ClinVar database (accession: SCV001433006).

## Ethics Statement

The studies involving human participants were reviewed and approved by the Comitato etico per la Sperimentazione Clinica (CESC) delle Province di Verona e Rovigo. The patients/participants provided their written informed consent to participate in this study.

## Author Contributions

AL, GM, and DG conceived the study. GM, FB, and AV collected the clinical data. AL, AC, AM, and AG carried out the experimental studies. EF and PB carried out the genetic study. AL and GM analyzed the data and wrote the manuscript. OO and DG critically revised the manuscript. All authors have approved the final version of the manuscript.

## Conflict of Interest

The authors declare that the research was conducted in the absence of any commercial or financial relationships that could be construed as a potential conflict of interest.

## Footnotes

1 www.hgmd.cf.ac.uk

2 http://wannovar.wglab.org/

3 www.jalview.org

4 http://molsim.sci.univr.it/marchetto/alas2/alas2info.php

5 http://molsim.sci.univr.it/marchetto/php/gene_detail.php?geneId=ALAS2#tabellaInit

6 http://www.mutationtaster.org/

7 http://genetics.bwh.harvard.edu/pph2/

8 https://sift.bii.a-star.edu.sg/

9 http://mutpred.mutdb.org/index.html

10 https://snps.biofold.org/snps-and-go/snps-and-go.html

11 http://www.cbs.dtu.dk/services/NetGene2/

12 https://www.fruitfly.org/seq_tools/splice.html

13 http://www.mutdb.org/mutpredsplice/about.htm
